# Advances and Challenges in Gene Therapy for Inherited Retinal Dystrophies: A Comprehensive Review

**DOI:** 10.7759/cureus.69895

**Published:** 2024-09-22

**Authors:** Raina Jain, Sachin Daigavane

**Affiliations:** 1 Ophthalmology, Jawaharlal Nehru Medical College, Datta Meghe Institute of Higher Education & Research, Wardha, IND

**Keywords:** crispr/cas9, gene editing, gene therapy, inherited retinal dystrophies, luxturna, retinal degeneration

## Abstract

Inherited retinal dystrophies (IRDs) are a diverse group of genetic disorders leading to progressive vision loss due to the degeneration of retinal photoreceptors. Gene therapy has emerged as a promising approach to address the underlying genetic causes of IRDs, offering the potential for restoring vision and halting disease progression. This review provides a comprehensive overview of gene therapy innovations for IRDs, focusing on the mechanisms, recent advancements, and ongoing challenges. We discuss the fundamental principles of gene therapy, including the use of viral and non-viral vectors, and highlight key developments such as the approval of Luxturna for RPE65-mediated retinal dystrophy and the application of gene editing technologies like CRISPR/Cas9. Despite these advancements, significant challenges remain, including vector delivery, long-term safety, and variable patient responses. This review also explores the future directions of gene therapy, emphasizing the need for further research to address these challenges and enhance therapeutic efficacy. By examining the current state of gene therapy for IRDs, this review aims to provide valuable insights into the potential for these treatments to transform the management of retinal diseases and improve the quality of life for affected individuals.

## Introduction and background

Inherited retinal dystrophies (IRDs) encompass a diverse range of genetic disorders characterized by the progressive degeneration of retinal photoreceptors, leading to significant vision impairment or blindness [1]. These conditions result from mutations in specific genes crucial for photoreceptor cells' normal functioning and survival. IRDs are broadly classified based on the pattern of retinal degeneration and the specific genetic mutation involved [1]. Key categories include Retinitis Pigmentosa (RP), a group of disorders leading to night blindness and loss of peripheral vision; Leber Congenital Amaurosis (LCA), which presents in infancy with severe vision loss; Stargardt Disease, the most common form of inherited macular degeneration affecting central vision; and Cone-Rod Dystrophy, where both cone and rod photoreceptors are impaired [2]. These conditions collectively affect approximately 1 in 4,000 individuals worldwide, with a profound impact on quality of life due to progressive vision loss, which can severely affect daily activities, employment, and overall well-being [3].

Gene therapy has brought a transformative shift in the management of IRDs. Historically, treatment options for these conditions were limited to symptomatic management and assistive devices. However, gene therapy represents a novel approach that addresses the genetic basis of IRDs [4]. Initial experiments in gene therapy for retinal diseases began in the 1990s, focusing on delivering functional copies of defective genes to retinal cells. Recent advancements have accelerated the development and application of gene therapy, exemplified by the approval of Luxturna (voretigene neparvovec) in 2017 for RPE65-mediated retinal dystrophy [5]. This milestone demonstrated the potential of gene therapy to restore vision in patients with specific genetic mutations. Ongoing clinical trials are expanding the scope of gene therapy, exploring various vectors, delivery methods, and gene targets, while innovations in gene editing technologies, such as CRISPR/Cas9, offer new possibilities for correcting genetic mutations [6].

This review aims to provide a comprehensive overview of gene therapy innovations for inherited retinal dystrophies. It will delve into the mechanisms underlying gene therapy, highlight recent advancements, and address the challenges and limitations faced in this field. By examining these elements, the review offers valuable insights into the future directions of gene therapy for IRDs and its potential to significantly improve the quality of life for individuals affected by these debilitating conditions.

## Review

Mechanisms of gene therapy for IRDs

Gene therapy involves introducing, removing, or altering genetic material within a patient's cells to treat or prevent disease. In the context of inherited retinal dystrophies (IRDs), the primary goals are to restore the function of defective genes, halt disease progression, and potentially reverse vision loss. This innovative approach has the potential to significantly enhance the quality of life for individuals affected by these debilitating conditions [7]. Gene therapy can be broadly categorized into two main types: gene replacement and gene editing. Gene replacement is typically used for autosomal recessive and X-linked disorders, where a functional gene copy is introduced to compensate for a defective one [8]. For example, gene replacement therapy for RPE65-related Leber congenital amaurosis aims to restore protein function. Gene editing techniques, such as CRISPR-Cas9, enable precise genome modifications, allowing for the correction of mutations at their source. This method is particularly promising for dominant disorders and may also involve gene silencing to mitigate the effects of harmful mutations [8]. Vectors are essential for effectively delivering therapeutic genes and can be classified into viral and non-viral types. Viral vectors, including Adeno-Associated Virus (AAV) and lentivirus, are commonly used in gene therapy for IRDs [9]. AAV is preferred due to its ability to infect both dividing and non-dividing cells without integrating into the host genome, thereby reducing the risk of insertional mutagenesis. AAV is suitable for smaller genes with a capacity of approximately 4.5 to 4.9 kb. In contrast, lentiviral vectors can integrate into the host genome, allowing for stable, long-term expression of the therapeutic gene. However, they primarily infect dividing cells and carry a risk of insertional mutagenesis, requiring careful consideration [9].

Non-viral vectors also contribute to gene therapy. Nanoparticles, for example, can encapsulate genetic material and deliver it to target cells, offering benefits such as reduced immunogenicity and improved targeting [10]. Electroporation, another non-viral method, uses electrical fields to increase cell membrane permeability, allowing for the direct introduction of genetic material into retinal cells. Advances in vector technology are crucial for improving the efficacy and safety of gene therapies [10]. Several genes are commonly associated with inherited retinal dystrophies, each vital to retinal function. The RHO gene, which encodes rhodopsin, is linked to retinitis pigmentosa (RP) and is associated with photoreceptor degeneration [11]. Mutations in the RPGR gene, essential for photoreceptor function, can lead to X-linked RP. Additionally, mutations in the BEST1 gene are associated with Best disease, affecting retinal pigment epithelial cells and resulting in vision loss [11]. Dysfunction in these genes typically produces non-functional or absent proteins, disrupting normal cellular processes in photoreceptors and retinal pigment epithelium (RPE) cells. This disruption ultimately causes cellular degeneration and progressive vision loss, highlighting the importance of targeted gene therapy to restore normal function and preserve vision [12]. Effective delivery methods are critical for the success of gene therapy in IRDs. Intraocular injection techniques are commonly used, with subretinal injection being the primary method for delivering therapeutic vectors [13]. This approach involves injecting the vector into the space between the retina and the RPE, providing direct access to photoreceptors. Although effective, this method is more invasive than intravitreal injection, which delivers the vector into the eye's vitreous humor. While intravitreal injection is less invasive and easier to perform, it may result in lower concentrations of the therapeutic agent reaching the targeted retinal cells [13]. The choice between systemic and localized delivery methods is also important. Localized delivery methods, such as subretinal injections, are preferred for targeting specific retinal cells, as they provide higher concentrations of the therapeutic agent directly where needed. In contrast, though less common for IRDs, systemic delivery may be used for conditions affecting multiple tissues or when localized delivery is not feasible [14]. Ongoing research and refinement of these delivery methods are essential for improving the efficacy and safety of gene therapies for inherited retinal dystrophies [14]. Innovations in gene therapy for inherited retinal dystrophies: mechanisms, challenges, and future perspectives are detailed in Table 1.

**Table 1:** Innovations in gene therapy for inherited retinal dystrophies: mechanisms, challenges, and future perspectives

Mechanism	Description	Examples in IRDs	Advantages	Challenges
Gene Replacement [15]	Introduction of a functional copy of a defective gene to restore normal function.	RPE65 gene therapy (e.g., Luxturna) for Leber congenital amaurosis.	Directly addresses the genetic cause of IRD, especially in monogenic disorders.	Long-term expression, immune response to viral vectors.
Gene Editing (CRISPR/Cas9) [16]	Editing of specific mutations within a gene to correct the defective gene sequence.	CRISPR/Cas9 was applied to correct mutations in RPGR and CEP290 associated with Retinitis Pigmentosa and Leber congenital amaurosis.	Potential for permanent correction of genetic defects.	Off-target effects, delivery efficiency, ethical concerns.
Gene Knockdown [17]	Silencing of a mutant gene to prevent the expression of harmful proteins.	RNA interference (RNAi) is used to reduce the expression of toxic proteins in diseases like Retinitis Pigmentosa, which is caused by gain-of-function mutations.	Suppresses harmful protein expression without altering the DNA sequence.	Incomplete knockdown, transient effects, off-target gene silencing.
Optogenetics [18]	Introduction of light-sensitive proteins to restore light perception in degenerated retinas.	Use of channelrhodopsins in patients with advanced Retinitis Pigmentosa.	Enables light perception in severely degenerated retinas where photoreceptors are non-functional.	There is limited resolution and color vision and the need for external devices (e.g., light goggles).
Exon Skipping [19]	Antisense oligonucleotides are used to skip mutated exons and allow the production of functional proteins.	Targeting specific mutations in genes like USH2A (Usher Syndrome) or CEP290.	Allows partial restoration of protein function in cases where a single exon mutation causes dysfunction.	It may not fully restore function, but it works best with specific types of mutations; multiple injections are required.
Gene Augmentation [20]	Insertion of multiple copies of a gene to increase the expression of functional protein in cases of haploinsufficiency.	Increasing expression of genes like BEST1 in Best Vitelliform Macular Dystrophy.	Boosts the production of essential proteins without altering the existing gene.	Delivery of multiple copies without triggering an immune response and precise control of expression levels.
Gene Suppression [21]	Blocking the expression of a mutant gene that produces toxic proteins.	Suppression of mutant rhodopsin gene (RHO) in Retinitis Pigmentosa to prevent degeneration of photoreceptors.	Prevents further degeneration in cases of toxic gain-of-function mutations.	Targeting only the mutant allele without affecting the wild-type allele increases the risk of incomplete suppression.
Stem Cell-Assisted Gene Therapy [22]	A combination of stem and gene therapy is needed to replace damaged retinal cells and introduce functional genes.	Using retinal progenitor cells combined with gene therapy in diseases like Age-Related Macular Degeneration (though not an IRD, a similar concept applies).	Provides both gene correction and cellular replacement for advanced cases of degeneration.	Complex technique requires precise control over gene expression and stem cell differentiation.

Innovations and advances

Gene therapy has achieved remarkable progress in recent years, particularly in treating inherited retinal dystrophies (IRDs). A notable breakthrough is the approval of Luxturna (voretigene neparvovec), which targets Leber congenital amaurosis caused by mutations in the RPE65 gene [23]. This therapy uses a recombinant adeno-associated virus (rAAV) vector to deliver a normal gene copy directly to retinal cells, effectively restoring gene function. In addition, numerous clinical trials are currently exploring gene therapies for various IRDs beyond RPE65-related conditions. These trials aim to establish the safety and efficacy of new treatments, expanding the range of genetic mutations that can be addressed and potentially offering hope to a broader patient population [24]. Advancements in gene editing have also been significant, with CRISPR/Cas9 emerging as a powerful tool for precise genetic modifications. This technology allows researchers to target specific DNA sequences for editing, enabling the correction of mutations directly in the genome. Recent developments include the first in vivo trials of CRISPR-based gene editing for conditions such as CEP290-mediated IRD [25]. Moreover, newer gene editing technologies offer enhanced precision and flexibility, including base editing and prime editing. Base editing enables the direct conversion of one DNA base to another without creating double-strand breaks. In contrast, prime editing combines CRISPR technology with reverse transcription to enable precise insertions, deletions, or replacements of DNA sequences. These advancements hold significant promise for improving the safety and efficacy of gene therapies [26]. Simultaneously, vector design improvements are crucial for gene therapy's success. Researchers are focused on enhancing the safety and efficiency of viral vectors commonly used to deliver therapeutic genes. Innovations include the development of targeted capsids that enhance the delivery of genes to specific retinal cell types, increasing the likelihood of therapeutic success [27]. There is growing interest in novel non-viral delivery systems, such as physical injection of naked DNA, aptamer-based systems, electroporation, gene guns, ultrasound, and magnetofection. These non-viral methods may offer cost-effective alternatives to traditional viral vectors and could improve the accessibility of gene therapies [27]. Innovations and advancements in gene therapy for inherited retinal dystrophies are detailed in Table 2.

**Table 2:** Innovations and advances in gene therapy for inherited retinal dystrophies

Category	Innovation/Advance	Description	Examples in IRDs	Impact/Significance
Approved Therapies [28]	Luxturna (RPE65)	The first FDA-approved gene therapy for IRDs was used to treat Leber Congenital Amaurosis (LCA) caused by RPE65 mutations.	Luxturna targets RPE65 gene mutations, restoring visual function in patients with LCA.	A milestone in gene therapy for IRDs, proving the feasibility and success of gene replacement therapy.
Gene Editing [29]	CRISPR/Cas9	Precise editing of defective genes to correct mutations, offering potential permanent treatment.	Trials for RPGR and CEP290 mutations associated with Retinitis Pigmentosa and LCA.	Revolutionizes treatment by allowing precise gene correction with the potential for lasting benefits.
New Gene Editing Tools [30]	Base Editing	A more refined gene-editing technique that allows precise nucleotide changes without causing double-strand breaks.	Potential for correcting single base mutations in RPGR, CEP290, and other genes involved in IRDs.	Increased precision in correcting mutations, reducing the risk of off-target effects.
Vectors [31]	AAV (Adeno-Associated Virus)	The most commonly used viral vector for delivering genes into retinal cells is known for its safety and long-term expression potential.	AAV vectors are used in Luxturna and various clinical trials for other IRDs, such as Usher Syndrome.	Reliable, safe vector for ocular gene therapy, leading to stable and efficient gene expression in retinal cells.
Improved Non-Viral Vectors [32]	Nanoparticles, Electroporation	Non-viral delivery methods that reduce the risk of immune responses and offer safer alternatives to viral vectors.	Early-stage trials exploring nanoparticle delivery for gene therapy in Retinitis Pigmentosa.	Lower immune response risk, potentially safer for repeated administration.
Delivery Methods [13]	Subretinal Injection	Direct injection into the subretinal space allows precise targeting of retinal cells.	Luxturna is administered via subretinal injection.	Effective delivery to retinal cells, although technically challenging and invasive.
Minimally Invasive Delivery [33]	Intravitreal Injection	Less invasive injection into the vitreous cavity allows easier delivery of gene therapies, though it limits reaching target cells in some diseases.	Being investigated for CRISPR-based therapies and AAV vectors.	Simplifies the procedure and reduces recovery time, though more research is needed to improve targeting efficiency.
Combination Therapies [34]	Gene Therapy + Stem Cell Therapy	Combining gene therapy with stem cell-based approaches to repair the genetic defect and restore damaged retinal cells.	Experimental approaches in conditions like Retinitis Pigmentosa and Age-Related Macular Degeneration (AMD).	Addresses both gene mutation and cellular damage, enhancing potential treatment efficacy.
New Technologies [35]	Prime Editing	An advancement over CRISPR/Cas9, offering precise and versatile editing capabilities without causing double-stranded DNA breaks.	It is not yet widely applied to IRDs but has the potential for diseases with specific genetic mutations.	Potentially safer and more precise than CRISPR, broadening the scope of treatable mutations.
Safety Improvements [36]	Immunomodulation Techniques	Development of strategies to mitigate immune responses against viral vectors, ensuring safer long-term application of gene therapies.	Trials incorporating immunosuppressive drugs or modulated vectors in gene therapy for IRDs.	Increases patient safety and ensures the longevity of gene therapy outcomes, particularly in repeat dosing situations.
Clinical Trial Expansion [37]	Broader Clinical Trial Enrollment Criteria	Expanding trials to include more diverse populations and earlier stages of disease, offering greater access to gene therapies.	There are ongoing clinical trials for RP, LCA, and Usher Syndrome with a wider array of enrolled patients.	Increases inclusivity and ensures treatments are effective across broader patient demographics and disease stages.
Regulatory Pathways [38]	Accelerated Approval Programs	Gene therapies for rare diseases like IRDs benefit from fast-tracked approval processes to expedite patient availability.	Luxturna's accelerated approval by the FDA set a precedent for future IRD gene therapies.	Shortens the timeline from bench to bedside, making life-changing therapies available to patients faster.

Challenges and limitations

Despite the promising advancements in gene therapy for inherited retinal dystrophies (IRDs), several challenges and limitations remain to be addressed [7]. A primary concern is the potential for side effects and immune responses. Viral vectors, commonly employed to deliver therapeutic genes, can trigger immune reactions in some patients. These immune responses may diminish the treatment's effectiveness and result in adverse effects that could compromise patient safety [36]. Furthermore, while short-term outcomes from gene therapy have been encouraging, the long-term effects and durability of these treatments are still largely unknown. Ongoing monitoring and long-term follow-up studies are essential to evaluate the sustainability of visual improvements and to identify any potential late-onset complications that may arise [36]. Technical and biological challenges also present significant hurdles in developing effective gene therapies for IRDs. Efficient delivery of therapeutic genes to the target retinal cells is crucial yet complex. Ensuring adequate transgene expression in the appropriate cells is vital for the therapy's success. Researchers are actively exploring improved delivery methods and enhanced targeting specificity to overcome these challenges [39]. Additionally, variability in patient response to gene therapy is a critical issue. Factors such as the stage of disease progression, genetic background, and environmental influences can contribute to differing outcomes among patients. Identifying predictive biomarkers and developing personalized treatment approaches may help address this variability and improve overall efficacy [39]. Ethical and regulatory considerations are also crucial in the context of gene therapy. Regulatory agencies continuously update guidelines as the field evolves rapidly to ensure new treatments' safety and efficacy. Navigating the regulatory landscape and meeting requirements for clinical trials and marketing approvals can be challenging and time-consuming [40]. Moreover, gene therapy raises important ethical concerns, including potential unintended consequences, the risk of genetic enhancements, and the need for equitable access to these innovative treatments. Ongoing discussions and collaborations among stakeholders, including patients, clinicians, researchers, and ethicists, are necessary to address these issues and ensure gene therapies' responsible development and application [40]. Table 3 outlines the challenges and limitations associated with gene therapy for inherited retinal dystrophies.

**Table 3:** Challenges and limitations in gene therapy for inherited retinal dystrophies

Category	Challenge/Limitation	Description	Impact	Potential Solutions
Safety Concerns [41]	Immune Response to Viral Vectors	Viral vectors, especially AAV, can trigger immune responses that limit therapy effectiveness and cause adverse reactions.	Risk of inflammation, reduced gene expression, or serious side effects.	Use of immunosuppressive drugs and development of less immunogenic vectors.
Efficacy Challenges [5]	Long-Term Durability	Uncertainty about the long-term expression and effectiveness of the introduced gene, particularly in rapidly degenerating retinal diseases.	Potential need for repeated treatments, risk of therapy failure over time.	Development of more durable vectors and strategies for sustained gene expression.
Delivery Limitations [42]	Targeting Specific Cells	Difficulty in precisely targeting the affected cells, particularly photoreceptors and retinal pigment epithelium.	Incomplete therapeutic effects if the gene is not delivered to the correct cells or if delivery is uneven.	Improving delivery methods, such as intravitreal or subretinal injections, or enhancing vector specificity.
Technical Hurdles [9]	Gene Size Limitations in Vectors	Some viral vectors, like AAV, have limited capacity for large genes, restricting their use for conditions involving larger genes.	Gene therapies may not be feasible for diseases with large genes (e.g., Usher Syndrome).	Exploring dual-vector systems or non-viral delivery systems like nanoparticles.
Patient Variability [43]	Heterogeneity in Genetic Mutations	Different mutations in the same gene can lead to varying phenotypes, complicating the development of one-size-fits-all therapies.	Reduced effectiveness or inapplicability of therapies across diverse patient populations.	Personalized gene therapies targeting specific mutations, gene-agnostic approaches.
Ethical Considerations [44]	Genetic Modifications and Gene Editing	Ethical concerns regarding permanent alterations to the human genome, especially with technologies like CRISPR/Cas9.	Hesitancy in clinical adoption, particularly for germline modifications and regulatory challenges.	Strict ethical guidelines focus on somatic cell therapy over germline editing.
Regulatory Barriers [45]	Approval and Accessibility	The regulatory approval process for gene therapies can be lengthy and expensive, particularly for rare diseases like IRDs.	Delayed access to potentially life-saving treatments for patients and high treatment costs.	Fast-track approval programs for rare diseases and increased investment in gene therapy research.
Cost and Accessibility [46]	High Treatment Costs	The cost of developing, manufacturing, and administering gene therapies is extremely high, limiting patient access, especially in low-resource settings.	Gene therapies may not be accessible to all patients, particularly in developing countries.	Developing more cost-effective production methods and exploring public funding or subsidies.
Biological Challenges [22]	Limited Treatment Window	Gene therapy is often most effective in the early stages of retinal degeneration, making it less effective for patients with advanced disease.	Late-stage patients may not benefit from the therapy, leading to a narrow treatment window.	Early diagnosis and intervention and the development of therapies suitable for advanced disease stages.
Potential Side Effects [47]	Off-Target Effects in Gene Editing	Technologies like CRISPR/Cas9 can cause unintended genetic changes, leading to potential harm or cancer risk.	Unpredictable effects that may complicate treatment or worsen the patient's condition.	Improving precision in gene editing tools and developing safer methods like prime editing.
Variable Patient Response [48]	Differential Efficacy Among Patients	Individual patient factors, such as age, disease progression, and immune system variations, can affect the efficacy of gene therapy.	Some patients may not respond to therapy or experience different benefits.	Tailored approaches based on individual patient characteristics, more personalized therapies.

Future directions

Gene therapy for inherited retinal dystrophies (IRDs) is advancing rapidly, driven by innovative technologies and approaches that pave the way for more effective treatments. Recent developments in gene editing, particularly through CRISPR technology, have introduced next-generation tools that enhance precision and minimize off-target effects [49]. Notable innovations include base editing, which enables the precise modification of single DNA bases without inducing double-strand breaks, and prime editing. This more advanced method allows for all base conversions, small deletions, and insertions without double-strand breaks. These techniques hold promise for correcting a broader range of genetic mutations, potentially expanding the treatable spectrum of IRDs [49]. The future of gene therapy is shifting towards personalized approaches, where treatments are tailored to the specific genetic makeup of each individual. This involves genetic profiling to identify specific mutations in a patient's genome and designing targeted therapies to address these unique genetic defects [50]. Combining gene editing with other modalities, such as RNA manipulation and CRISPR-based transcriptional regulation, can enhance therapeutic outcomes and provide multifaceted treatment strategies for complex genetic disorders [50]. Integrating gene therapy with pharmacological or surgical interventions may enhance efficacy and improve patient outcomes [51]. For instance, utilizing drugs that support or amplify the effects of gene therapy, such as those promoting retinal health or protecting against degeneration, can lead to better functional recovery and long-term vision restoration. Additionally, combining gene therapy with surgical approaches, like retinal implants or cell replacement therapies, may offer comprehensive solutions for restoring vision in patients with advanced IRDs [51]. Stem cell therapy represents another promising avenue for enhancing gene therapy. By combining gene editing techniques with stem cell transplantation, it may be possible to restore or replace lost or dysfunctional retinal cells, providing a dual approach to treating IRDs. This synergy could improve functional recovery and long-term vision restoration [52]. Translational research is crucial for advancing gene therapies from the laboratory to clinical applications. Developing and utilizing animal models that accurately replicate human IRDs is essential for testing the efficacy and safety of new therapies before conducting human trials [53]. Navigating the complex regulatory landscape to ensure new therapies meet safety and efficacy standards for clinical use is also critical. Addressing research gaps, such as expanding studies on genetic heterogeneity and improving patient recruitment and access, will further advance gene therapy for IRDs [53]. Future directions in gene therapy for inherited retinal dystrophies are detailed in Table 4.

**Table 4:** Future directions in gene therapy for inherited retinal dystrophies

Category	Future Direction	Description	Potential Impact	Challenges
Next-Generation Gene Editing [35]	Prime Editing	A precise and flexible gene editing technique allows for correcting genetic mutations without causing double-strand DNA breaks.	More accurate and safer gene therapy for correcting mutations in IRDs, expanding the range of treatable conditions.	Development for clinical use, delivery of prime editors to target cells efficiently.
Personalized Gene Therapy [54]	Patient-Specific Therapies	Tailoring gene therapies to the genetic profile of individual patients or specific mutations.	Increased efficacy and reduced adverse effects by targeting specific genetic defects.	Cost and complexity of creating personalized therapies, regulatory hurdles.
Combination Therapies [55]	Gene Therapy + Pharmacological or Surgical Approaches	Combining gene therapy with other interventions like pharmacological agents or retinal implants for synergistic effects.	Enhanced treatment outcomes, especially in cases where gene therapy alone is insufficient.	Integration of different therapeutic modalities and complex clinical trials to assess efficacy.
Stem Cell Integration [22]	Gene Therapy + Stem Cell Therapy	Using stem cells to deliver gene therapy and regenerate damaged retinal cells simultaneously.	Offers a dual approach to repair genetic defects and replace damaged tissue, especially in late-stage IRDs.	Difficulties in controlling stem cell differentiation and ensuring safety and risk of immune rejection.
Improved Vectors [56]	Safer and More Efficient Delivery Vectors	Development of new viral and non-viral vectors that offer enhanced safety, targeting specificity, and gene delivery efficiency.	Reduces immune response risks, allows for more precise targeting of retinal cells, and improves gene expression longevity.	Engineering vectors that balance safety and efficiency while applying to a wide range of patients.
Non-Viral Delivery Systems [57]	Nanoparticles and Other Non-Viral Vectors	Advancements in non-viral delivery systems, such as nanoparticles, liposomes, and electroporation methods to deliver genes without using viruses.	Potentially safer alternatives to viral vectors, reducing immune responses and expanding the range of diseases treatable with gene therapy.	Ensuring efficient and sustained gene delivery, optimizing targeting and uptake by retinal cells.
Gene Therapy for Complex Diseases [58]	Targeting Polygenic and Multifactorial Diseases	Expanding gene therapy beyond monogenic IRDs to target diseases with multiple genetic and environmental components (e.g., Age-related Macular Degeneration).	Broadens the scope of gene therapy to treat more complex ocular diseases that affect a larger patient population.	Challenges in identifying all genetic factors contributing to complex diseases and difficulty in designing therapies that address all relevant factors.
Advanced Diagnostic Tools [59]	Early Detection and Biomarker Identification	Advanced imaging technologies and genetic tests have been developed to identify patients who may benefit from gene therapy earlier in the disease progression.	It increases the effectiveness of gene therapy by targeting the disease at earlier, more treatable stages and allows for better patient selection in clinical trials.	High cost of developing and implementing new diagnostic technologies, access to early diagnosis in lower-resource settings.
Long-Term Monitoring and Safety [60]	Long-Term Follow-Up Studies and Safety Monitoring	Establishing robust long-term monitoring systems to track the safety and efficacy of gene therapy over time, addressing concerns about durability.	Ensuring that gene therapies have lasting effects without causing harmful side effects improves trust and confidence in gene therapy for patients and clinicians.	Cost and logistics of conducting long-term follow-up studies, ensuring patient adherence to follow-up protocols.
Global Accessibility [61]	Affordable and Accessible Gene Therapy	Research and strategies to reduce the cost of gene therapy and make it available to patients in low-resource settings.	Expands access to life-changing treatments for patients worldwide, particularly in developing countries where IRDs may be more prevalent.	Reducing manufacturing costs, creating distribution channels in low-resource regions, and overcoming regulatory hurdles in different countries.
Regulatory and Ethical Frameworks [62]	Harmonized Global Regulatory Standards	Establishing consistent global regulatory standards and ethical guidelines for gene therapy, especially concerning genetic modifications.	Streamlines the approval process for gene therapies, ensures ethical considerations are met across different countries, and accelerates patient access to approved treatments worldwide.	Ensuring international cooperation on regulatory standards, addressing varying ethical perspectives on gene therapy across different cultures.

## Conclusions

In conclusion, gene therapy for inherited retinal dystrophies has made remarkable progress, exemplified by breakthroughs like Luxturna and advancements in gene editing technologies such as CRISPR/Cas9. However, significant challenges remain. For instance, viral vectors like Adeno-Associated Virus (AAV), commonly used in ocular gene therapies, pose risks of immunogenicity and have limited capacity for larger genes, while non-viral vectors, such as nanoparticles, still require optimization for effective targeting of specific retinal cells. Additionally, safety concerns related to off-target effects in gene editing present long-term risks, including unintended genetic changes that could potentially lead to tumorigenesis. While Luxturna has paved the way for gene therapy in retinal diseases, it is important to highlight other promising therapies in clinical trials, such as those targeting X-linked retinitis pigmentosa (RPGR) and Leber congenital amaurosis (CEP290), to provide a broader view of future treatments. Ethical and regulatory issues, particularly concerning gene editing technologies like CRISPR, must also be considered. The potential for permanent genetic modifications raises ethical concerns, especially regarding germline editing, and the regulatory framework must evolve to ensure the safe and equitable use of these therapies, addressing challenges related to cost and accessibility. While this article focuses on retinal diseases, the advancements in gene therapy have broader implications for other genetic disorders, such as muscular dystrophies and hemophilia, where similar therapeutic approaches could be applied. Most importantly, the patient-centered perspective must be emphasized. Early clinical trials are demonstrating real-world improvements in visual acuity and functional vision, offering new hope to patients with previously untreatable conditions. However, more research is needed to assess the long-term outcomes of these therapies and their impact on patients' quality of life. By addressing these challenges and expanding the scope of therapies, gene therapy has the potential to revolutionize not only retinal disease treatment but also the management of other genetic conditions.
